# Does Basic Psychological Need Satisfaction Matter to College Students’ Sustained Volunteering? A Mixed-Methods Study

**DOI:** 10.3390/ijerph182413229

**Published:** 2021-12-15

**Authors:** Shuang Zheng, Meilin Yao, Lifan Zhang, Jing Li, Huilin Xing

**Affiliations:** Faculty of Psychology, Beijing Normal University, Beijing 100875, China; zspsy@mail.bnu.edu.cn (S.Z.); zhanglifan@mail.bnu.edu.cn (L.Z.); 201831061007@mail.bnu.edu.cn (J.L.); 201821061091@mail.bnu.edu.cn (H.X.)

**Keywords:** college student volunteer, basic psychological need satisfaction, sustained volunteering, mixed methods

## Abstract

Based on the self-determination theory (SDT), this study used a mixed-methods (i.e., quantitative and qualitative approaches) design to explore the role of basic psychological need satisfaction (BPNS) played in sustained volunteering. Quantitative analysis of 803 college student volunteers revealed that competence and relatedness need satisfaction had significant associations with sustained volunteering, while autonomy need satisfaction did not. Furthermore, latent profile analyses identified five profiles of BPNS: low (Profile 1), relatively low (Profile 2), moderate (Profile 3), low autonomy-high competence and relatedness (Profile 4), and high (Profile 5). Volunteers in Profile 4 and Profile 5 reported higher sustained volunteering than those in other profiles. Subsequent qualitative synthesis of interview data from 33 college student volunteers found that competence need satisfaction (45.58%) was mentioned most frequently among the factors promoting sustained volunteering, then followed by relatedness (27.43%) and autonomy need satisfaction (11.06%). These findings highlight the important role of BPNS, especially competence and relatedness need satisfaction, in promoting college students’ long-term volunteering.

## 1. Introduction

Volunteering is a planned, long-term, and nonobligatory prosocial activity [1]. It has been found crucial not only to foster social and economic integration [2,3], but also to the considerable psychological and social benefits for volunteers themselves, such as developing relationships with other volunteers, learning new skills, being productive and creative, and feeling self-efficacy [4,5,6]. However, how to facilitate the sustainability of volunteering has become a pressing issue as volunteering rates are constantly decreasing while rates of drop-out, nonperformance, and non-attendance of volunteers are increasing [3,7,8,9]. Therefore, identifying the facilitative factors of sustained volunteering may provide insight into why people continue volunteering over an extended period.

Sustained volunteering, a planned and recurring voluntary activity, requires a long-term commitment to service [7,10,11]. Research has shown service length and frequency (i.e., objective indicators) [10] as well as volunteer retention (i.e., subjective indicators) [12,13] are two common indicators to measure sustained volunteering. Thus, in the current study, sustained volunteering was measured by both objective and subjective indicators. Several researchers using a variable-centered approach (e.g., regression analysis, structural equation modeling) studied the antecedents of sustained volunteering based on the self-determination theory (SDT) [14,15,16] and proposed that basic psychological need satisfaction (BPNS) (i.e., autonomy, competence, relatedness) was a critical factor predicting retention of volunteers [4,12,13]. However, previous research results regarding the relative importance of each need satisfaction in predicting sustained volunteering were neither clear nor consistent, and most of the research was conducted in Western contexts [13]. Both inconsistent results and limited cultural contexts suggest a need to deeply explore the relationships between three BPNS and sustained volunteering, especially in the Chinese context in which volunteer service activities are just in the primary development stage. More importantly, considering college students constitute the majority of volunteers in China, especially in terms of the Chinese context, by 2018, more than 270,000 college students had been involved in volunteering in 22 regions in Central and Western China, promoting the development of local education, health, and poverty alleviation [17]. However, Yao and Guo [18] found less than one-third of the volunteer activities in which some college students participate were continuous or regular, while most of the others were temporary. It is necessary to explore the factors influencing the sustainability of Chinese college students’ volunteering.

Furthermore, to our best knowledge, few studies had considered the various possible combinations of the three BPNS (e.g., low autonomy combines with high competence and relatedness) among a volunteer sample and then investigated their effects on sustained volunteering from a person-centered perspective. In addition, few studies explored the relationships between three BPNS and sustained volunteering using qualitative methods or combining both quantitative and qualitative data (i.e., mixed methods). These research gaps may hinder a deeper understanding of the nature and effects of three BPNS in the field of volunteering.

Consequently, to address the above research gaps, the present research aimed to deeply explore the role of three BPNS in sustained volunteering by using mixed methods (i.e., quantitative and qualitative approaches). In the quantitative phase, by taking both variable- and person-centered perspectives, the current study aimed to (1) explore the relationships between volunteers’ three BPNS and sustained volunteering from a variable-centered perspective; (2) identify the profiles in terms of the BPNS by using latent profile analyses (LPA), and (3) examine the relationships between these profiles and sustained volunteering from a person-centered perspective. In the qualitative phase, a semistructured interview was conducted to further explore whether the satisfaction of three psychological needs was an important factor promoting sustained volunteering and discussed the relative importance of three BPNS.

## 2. Literature Review and Theoretical Framework

### 2.1. Self-Determination Theory in Volunteering

SDT postulates the existence of three basic psychological needs: autonomy, relatedness, and competence [14,15,16]. The need for autonomy is defined as the need to experience volition and willingness during an activity. The need for relatedness refers to the desire to feel connected, supported, or cared for by other people and maintain relationships with others. The need for competence concerns the need to feel effectiveness and mastery in performing activities. As the Basic Needs Satisfaction in General Scale (BNSG) developed by Johnston and Finney [19] was widely used, the current study used this scale to measure the individual’s three BPNS. According to SDT, the satisfaction of these three basic psychological needs through a behavior (such as volunteering) plays a central role in facilitating the internalization of the behavior and turning it into a sustained intention to perform the behavior [13,20]. A body of research based on the SDT has shown that satisfaction of the three basic psychological needs is associated with a variety of positive volunteering-related outcomes, including volunteer engagement [13,21], affective commitment [22], volunteer satisfaction [12,23], and life satisfaction [24]. Thus, volunteers’ three BPNS seems to be a valuable resource for understanding intentions of sustained volunteering within the framework of SDT.

### 2.2. Basic Psychological Need Satisfaction and Sustained Volunteering

To date, both variable- and person-centered approaches are available for examining the association between three BPNS and sustained volunteering. Theoretically, the variable-centered approach implicitly assumes that all volunteers are inherently homogeneous and focuses on the average association variables across the whole sample [25,26]. Using this approach, we can understand which BPNS are stronger predictors of sustained volunteering. In addition, the person-centered approach assumes that volunteers are inherently heterogeneous and focuses on identifying subpopulations characterized by distinct profiles with varying satisfaction of the three needs. This approach is naturally suited to the consideration of the joint effects of three BPNS combinations. Both variable-centered and person-centered approaches can generate their unique insights about the associations between three BPNS and sustained volunteering respectively.

#### 2.2.1. From a Variable-Centered Perspective

A number of studies using a variable-centered approach (such as regression analysis, structural equation modeling) have investigated the potential association between the satisfaction of the three basic psychological needs and sustained volunteering from an integrated perspective. Some studies have found that satisfaction of the three basic psychological needs experienced through volunteering played a vital role in individuals’ continuing efforts on sustained volunteering or their future volunteering intentions [23,27]. However, some researchers have argued that only one or two basic psychological needs satisfaction are related to sustained volunteering, or only a certain kind of need plays the greatest role in promoting sustained volunteering. For example, the satisfaction of the needs for competence and relatedness, rather than autonomy, has been found to positively predict American high school students’ intentions to participate in more community-based service-learning projects in the future [28] and Australian employees’ affective commitment to corporate volunteering [22]. Researchers also found that satisfaction of the needs for autonomy and relatedness was the main index for general need satisfaction in a sample of Korean Olympic volunteers [23] and was the critical determinant of intentions to remain for Dutch adult volunteers (mean age 44.6 years) [12]. Additionally, some researchers found that satisfaction of the need for relatedness was an important predictor for British undergraduate students’ intentions and interest in volunteering [29] and the continued volunteering of Americans for an organization [27]. While other researchers found that satisfaction of the relatedness need was not related to Romanian volunteers’ work engagement and intentions to quit when controlling for two other needs satisfaction [13]. Therefore, conclusions regarding the relative importance of each BPNS in predicting sustained volunteering are neither clear nor consistent. These inconsistent results may be caused by cultural and group differences in the samples.

Most of the studies on sustained volunteering have been conducted in Western contexts; very few have been situated in Eastern cultures, particularly in China [13,27], which might produce some cultural bias limiting the generalizability of their findings. Scholars have argued that cross-cultural differences might have cultural influences on individuals’ motivations for volunteering [30,31]. For instance, Eastern cultures like Chinese culture, are more collectivistic than Western cultures [32]. People in collectivistic societies characterized by cohesive groups emphasize group identification and maintaining harmonious interpersonal relationships, while individualistic cultures emphasize personal autonomy and self-realization [31,33]. In addition, Finkelstein [30] demonstrated that individualism was closely associated with self-oriented motives (such as career-related volunteer objectives), whereas other-oriented motives (such as the desire to strengthen social ties) were closely linked with collectivism. Hence, we speculate that building good relationships may be an important part of volunteer satisfaction in Chinese culture, while Western volunteers may regard personal gain (e.g., personal growth and development) as a crucial source of satisfaction [34]. Hence, research results from Western cultures on the role of the three types of BPNS in sustained volunteering may be inappropriate to apply directly to the Chinese context. Additionally, three BPNS may have different effects for different groups. For college students, they seek to learn necessary skills and enhance their competence through volunteer activities to help them enter the labor market [35]. Thus, satisfying their competence need in volunteering may be critical to improving their intentions to participate in subsequent volunteering.

In summary, it is important to explore the complicated effects of autonomy, competence and relatedness need satisfaction in sustained volunteering for Chinese college students, which may provide insight into how to boost the ongoing participation of Chinese college students in volunteering. In the present research, we treated BPNS as three separate factors (i.e., autonomy, relatedness, and competence needs satisfaction) [4] and respectively investigated their relationships with sustained volunteering using a variable-centered approach. Overall, from the variable-centered perspective, the first aim of the current study was to investigate how the three BPNS related to sustained volunteering among Chinese college volunteers. We proposed the following hypothesis:

**Hypothesis** **1.** *Compared with autonomy need satisfaction, competence and relatedness needs are stronger predictors of sustained volunteering for Chinese college volunteers*.

#### 2.2.2. From a Person-Centered Perspective

Studies adopting the variable-centered approach have provided valuable insights into the relationships between BPNS and sustained volunteering. However, the variable-centered approach doesn’t consider the combined effects of three BPNS or the interplay among the three BPNS. For instance, volunteers may feel closely connected to others but lack autonomy and voice in volunteering [36]. The person-centered approach, such as LPA, is useful for filling this gap. Because it allows for the identification of distinct subpopulations of volunteers that are characterized by distinct profiles concerning three BPNS. The key difference between the two approaches is that the variable-centered approach decomposes the covariances to highlight relationships among the variables, whereas the person-centered approach decomposes the covariances to highlight relationships among individuals [25,26]. Moreover, one of the benefits of the person-centered approach focuses on the combined effects of the three BPNS rather than the isolated effects of each separate one. Furthermore, it allows one to investigate the complex interactions among the three BPNS, which would be hard to trace with a variable-centered approach [25,26]. Therefore, in the current study, in addition to a variable-centered approach, we would adopt a person-centered approach to investigate what types of profiles exist among Chinese college students and how the profiles relate to sustained volunteering.

Although there are few studies directly investigating BPNS profiles in volunteering, some research from other areas may provide meaningful information for the current study. Some studies have examined BPNS profiles in different contexts (e.g., education, work, social networking domain, etc.) across different samples (e.g., teachers, workers, students) and their relations to various outcomes (e.g., educational, emotional) [37,38]. Researchers have identified two similar profiles: one has high satisfaction of the three basic psychological needs and the other has low satisfaction of these needs. The former was associated with the most desirable outcomes, including high levels of personal growth [39], self-determined motivation [40], and well-being [41], while the latter was found to score the highest on maladaptive outcomes, including negative emotion, such as anxiety [36] and high levels of dropout intentions [38]. Studies also identified several additional BPNS profiles, including but not limited to high competence [41], low-moderate need satisfaction (low autonomy and competence coupled with moderate relatedness) [42], and average need satisfaction [43].

Although the person-centered research has provided some understanding of the three BPNS profiles, we still need to identify the three BPNS profiles in volunteering contexts, especially among Chinese college student volunteers and, reveal the possible associations of these profiles with sustained volunteering. Thus, from the person-centered perspective, the second aim of the current study was to investigate the latent profile types of Chinese college volunteers’ three BPNS and examine the relationships between these profiles and sustained volunteering. We proposed the following hypotheses:

**Hypothesis** **2.** *Volunteers’ three BPNS are best represented by a limited number of profiles*.

**Hypothesis** **3.** *Different profile types differ in sustained volunteering*.

#### 2.2.3. Mixed-Methods Approach

Although previous variable- and person-centered research has provided some understanding of the relationships between the three BPNS and sustained volunteering, most research relied on quantitative data and generally ignored qualitative data on volunteers (such as semistructured interviews). As Reznickova and Zepeda [44] noted, semistructured interviews could allow volunteers to freely express their opinions and feelings by narrating their volunteering experiences and provide opportunities to ask exploratory questions and follow up on topics. Only using quantitative analysis may not be sufficient to fully understand the contributions of the three BPNS to sustained volunteering, but combining it with qualitative analysis may provide a full picture. In the current study, therefore, we also investigated whether the satisfaction of the three psychological needs is a key factor facilitating sustained volunteering and explored the relative importance of the satisfaction of the three needs through semistructured interviews. Based on SDT, we would design the interview questions according to the method used by Podlog and Dionigi [45] and the results of the quantitative phase. For instance, we interviewed participants about autonomy need satisfaction (e.g., “Did you participate in volunteering initiatively? Please describe it specifically.”), to explore how autonomy need satisfaction could influence the sustainability of volunteering.

In the qualitative phase, we aimed to gain a more comprehensive understanding of the relationships between three BPNS and sustained volunteering. Due to the study’s exploratory nature, there was no specific hypothesis on the results of qualitative analysis.

## 3. The Current Study

The present study adopted a mixed-methods (i.e., quantitative and qualitative approaches) design to deeply explore the associations between the three BPNS and sustained volunteering among Chinese college student volunteers. Two different methodologies (i.e., quantitative and qualitative) were used.

First, in the quantitative phase, by taking both variable- and person-centered perspectives, a total of 803 college student volunteers completed a questionnaire survey. From the variable-centered perspective, we investigated how the three BPNS related to sustained volunteering. From the person-centered perspective, we investigated the latent profile types of volunteers’ three BPNS with LPA and examined the various representations of different latent profiles of three BPNS in sustained volunteering.

Second, to elaborate the role of the three BPNS in promoting sustained volunteering, in the qualitative phase, we recruited again 33 college student volunteers to participate in a semistructured interview. These participants were not the samples used in the quantitative phase. This phase helped explain and understand the findings from the quantitative phase and provided valuable information for establishing a long-term mechanism for volunteering.

## 4. Methods in Quantitative Phase

### 4.1. Participants and Procedure

Volunteers were recruited from training seminars in large-scale volunteer activities. A total of 803 college student volunteers (588 female, 213 male, and 2 with missing gender information) were asked to complete the questionnaires. The participants’ average age was 21.64 years old (SD = 1.63). The types of volunteer activities participants engaged in the past year included: volunteer teaching (supporting education in rural and poverty-stricken areas, etc.), helping socially vulnerable groups (the elder, disabled or autistic children, etc.), supporting various activities (sports events, vocal concerts and various international meetings, etc.), environmental improvement (tree planting, environmental cycling, etc.), community activities (snow cleaning, legal popularization, etc.), and other voluntary work. The institutional review board (IRB) of the authors’ institution approved all the measurements and procedures for the study. The first page of the questionnaire clarified the purpose of the study and the rights of participants.

### 4.2. Measures

We adapted the Basic Needs Satisfaction in General Scale (BNSG) revised by Johnston and Finney [19] to the volunteer context by substituting the words “my life” with “my volunteer work” or “my volunteering activities”. In total, this scale consisted of three subdimensions, namely, autonomy (3 items; e.g., “I feel like I am free to decide for myself how to do my volunteer work”), relatedness (7 items; e.g., “I get along with people I come into contact with in my volunteering activities”), and competence (6 items; e.g., “Recently, I have been able to learn interesting new skills through volunteering activities”. Items were rated on a 7-point Likert scale (1 = Not at all true, 7 = Very true). High scores indicated high levels of BPNS. The results of confirmatory factor analysis indicated that the original factor structure of the BNSG provided a good fit to the data: *χ*^2^ (51) = 150.456, *p* < 0.001, *χ*^2^/*df* = 2.95, CFI = 0.93, and RMSEA [90% CI] = 0.08 [0.07, 0.10]. In the present study, Cronbach’s alpha reliability coefficients for autonomy, relatedness, competence and total scale were 0.81, 0.64, 0.89, and 0.85, respectively.

Sustained volunteering was composed of three indicators [10,12,13]: service length, service frequency, and intent of volunteers to remain with the volunteer organization. Service length and frequency were the objective indicators [10], while intent to remain was the subjective indicator [12,13]. Service length measured the time (i.e., hours) they had spent on volunteer work during the last year. The variable was coded with the midpoints of the original answer: 0 meant the respondent did not volunteer at all in the last year, 10 meant volunteered less than 20 h, 30 meant 20 to 39 h, 60 meant 40 to 79 h, 120 meant 80 to 159 h, and 200 meant 160 h or more [46]. To assess service frequency, we employed a 6-point measure and asked the volunteers to indicate how often they had performed volunteer work during the last year (1 = never; 2 = once; 3 = a few times; 4 = almost every month; 5 = almost every week; 6 = almost every day). The score of objective indicators was the average of standard scores of the two factors above. For the intent of continuing to volunteer (i.e., the subjective indicator), we asked the volunteers to rate three items (e.g., “I plan to continue my volunteer work for the next six months”) on a 7-point scale (1= totally disagree, 7 = totally agree). Cronbach’s alpha was 0.64.

### 4.3. Statistical Analysis

Analyses were conducted via the software of SPSS 20.0 (IBM Corp, Armonk, NY, USA) and Mplus 8.0 (Mplus team, Los Angeles, CA, USA) [47]. There were no missing data in the study except for gender. In the follow-up analysis, gender and age were used as control variables. For the variable-centered analyses, we investigated the relationships between the three BPNS and sustained volunteering by using correlation analysis and regression analysis. For the person-centered analyses, two procedures were used to analyze our data. In the first procedure, LPA was conducted to identify the latent profiles of the three BPNS using Mplus 8.0. Three indicators were standardized [43]. To determine the optimal number of latent profiles, 1 to 5 profile solutions were evaluated and compared based on fit statistics, interpretability, and theoretical considerations. To do so, the following statistical fit indices were used: the Akaike information criteria (AIC), Bayesian information (BIC) and Adjusted Bayesian information (aBIC), entropy, the Lo–Mendell–Rubin likelihood ratio test (LMRLRT), the Lo–Mendell–Rubin adjusted likelihood ratio test (ALMRLRT), and the bootstrap likelihood ratio test (BLRT). In the second procedure, to examine the potential differences among those profiles in sustained volunteering, we used multivariate analysis of variance (MANOVA).

## 5. Results in Quantitative Phase

### 5.1. Analysis Results of Variable-Centered

Supplementary Table S1 presents the means, standard deviations, and correlations analysis among the variables. For three BPNS, autonomy was significantly correlated with relatedness (*r* = 0.27, *p* < 0.01), competence (*r* = 0.24, *p* < 0.01), and general need satisfaction (*r* = 0.76, *p* < 0.01). Relatedness was significantly correlated with competence (*r* = 0.80, *p* < 0.01) and general need satisfaction (*r* = 0.79, *p* < 0.01). Competence was significantly correlated with general need satisfaction (*r* = 0.78, *p* < 0.01). Relatedness (*r* = 0.16, *p* < 0.01; *r* = 0.47, *p* < 0.01), competence (*r* = 0.19, *p* < 0.01; *r* = 0.46, *p* < 0.01) and general need satisfaction (*r* = 0.14, *p* < 0.01; *r* = 0.39, *p* < 0.01) were also significantly correlated with objective and subjective indicators of sustained volunteering, respectively. But, autonomy need satisfaction was only significantly correlated with the subjective indicator of sustained volunteering (*r* = 0.09, *p* < 0.01). In addition, the correlations between gender and age and other variables were significant (*p* < 0.05), so they were controlled in the follow-up analysis.

The multivariate linear regression analyses were performed to test our hypotheses. As shown in Table 1, after controlling for gender and age, only competence need satisfaction was positively related to service length, service frequency, and objective indicators of sustained volunteering. Both competence and relatedness need satisfaction were positively related to subjective indicators of sustained volunteering. The association between autonomy and both indicators of sustained volunteering was not significant.

### 5.2. Analysis Results of Person-Centered

#### 5.2.1. Profiles of Three BPNS

The results of five models (from one- to five-profile patterns) are presented in Supplementary Table S2. In models two to five, AIC, BIC and aBIC continued decreasing, and the changes in these indicators gradually became slight around three profiles. The five-profile model yielded optimal model values because it had lower AIC, BIC, and aBIC values than the other model, as well as acceptable entropy values. Furthermore, the LMRLRT and ALMRLRT values indicated that the five-profile model was better than the four-profile model, suggesting that the four-profile model should be excluded. Moreover, the five-profile model identified one distinct profile characterized by a low level of autonomy and high levels of competence and relatedness. This result enriches our knowledge of the relationships between profiles and sustained volunteering. In particular, it verifies the unique role of competence and relatedness need satisfaction in sustained volunteering.

**Figure 1:**
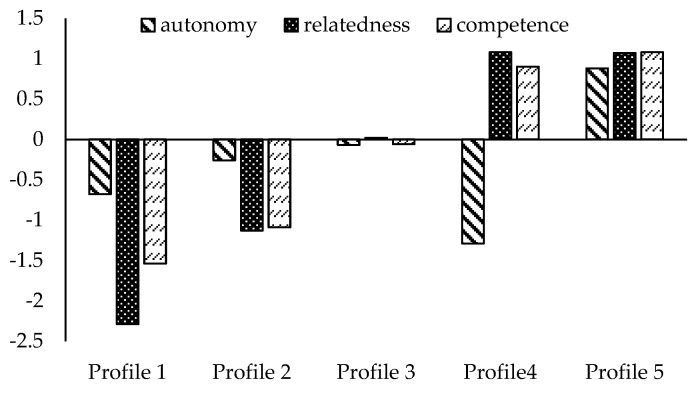
Basic psychological need satisfaction profiles of volunteers (Note. Scores for all three indicators were standardized).

As depicted in Figure 1 and Table 2, MANOVA revealed that these five profiles significantly differed in autonomy, relatedness and competence need satisfaction. Profile 1 (*n* = 37, 5%) was characterized by low levels of satisfaction for each need, we labeled it the Low Group. Profile 2 (*n* = 172, 21%) was characterized by relatively low levels of satisfaction of the three needs; we labeled it the Relatively Low Group. Profile 3 (*n* = 340, 42%) was characterized by moderate levels of satisfaction of the three needs; we labeled it the moderate group. Profile 4 (*n* = 60, 7%) was characterized by a low level of autonomy and high levels of competence and relatedness; we labeled it the Low Autonomy-High Competence and Relatedness Group. Profile 5 (*n* = 194, 24%) was characterized by high levels of satisfaction of all three needs; we labeled it the High Group.

#### 5.2.2. Profiles Differences in Sustained Volunteering

The five profiles were compared based on their levels of sustained volunteering (see Table 2). After controlling for gender and age, MANOVA revealed significant effects of profile membership on sustained volunteering. The Tukey post hoc tests revealed that the low autonomy-high competence and relatedness group had longer service length than the low group, the relatively low group, and the moderate group. The high group had a longer service length and higher service frequency than the relatively low group. For the objective indicators of sustained volunteering, both the low autonomy-high competence and relatedness group and the high group scored higher than the relatively low group. For the subjective indicators of sustained volunteering, all the groups were significantly different, except that there was no significant difference between the low autonomy-high competence and relatedness group and the high group.

Briefly, volunteers in the low autonomy-high competence and relatedness group and the high group reported equal but higher sustained volunteering than those in other profiles.

## 6. Methods in Qualitative Phase

In the quantitative phase, variable- and person-centered approaches were adopted simultaneously to understand the relationships between the three BPNS and sustained volunteering among college student volunteers. The data obtained from the interviews in the qualitative phase will hopefully provide a more comprehensive picture of the role of the three BPNS in sustained volunteering.

### 6.1. Participants and Procedure

The participants were recruited from a website of a university in Beijing, China. A total of 33 college students with volunteer experience participated in the semistructured interview. These participants were not the samples used in the quantitative phase. The mean age of the participants was 21.55 ± 0.60 years old, including 11 females and 22 males. All of the participants had performed voluntary service in the past year. The measures of service frequency and length used in the qualitative phase were the same as those in the quantitative phase. To be specific, the average service frequency of the participants was 3.24 ± 0.19 times (between “a few times” and “almost every month”), and the participants’ average service time was 36.97 ± 8.25 h. The types of volunteer activities participants engaged in were similar to those in the quantitative phase.

The investigator completed the one-on-one interviews in the laboratory. Each face-to-face interview took approximately 15–20 min. After completing the interview, each participant received 20 RMB. The procedures were approved by the Institutional Review Board (IRB) of the authors’ institution. All the participants completed the informed consent forms.

### 6.2. Interview Questions

The results of the quantitative phase showed that the three BPNS played different roles in predicting sustained volunteering. Based on these results and the method used by Podlog and Dionigi [45], the current research designed the interview questions focused on the volunteers’ three BPNS, as well as their relationships with sustained volunteering. The interviews were semistructured to conduct multiple probes and ask follow-up questions. We adapted the interview questions from Podlog and Dionigi [45] to suit the volunteer context. For instance, we substituted the sentence “what do you feel you have gained from the training experience?” with “how do you evaluate what you have gained from volunteering?” to probe competence need satisfaction. The comprehensibility of interview questions was tested with three volunteers who did not involve in the qualitative phase.

In the interviews, the volunteers were asked: “What are the reasons for you to continue volunteering? Please give specific examples. (Q1)”. After the participant’s narration (e.g., one of the participants stated, “when I was a volunteer commentator in the museum, I felt that I had gained knowledge. In addition, it’s amazing to meet many kinds of people when explaining cultural relics”), follow-up questions were asked, aiming to investigate their perceptions of the three BPNS, including the following: autonomy (e.g., “Did you participate in volunteering initiatively? Please describe it specifically. (Q2)” and “Did you have opportunities to make your own decisions about the volunteer work you did? Please give specific examples. (Q3)”), competence (e.g., “Do you think you are good at the volunteer work you participated in? Why? (Q4)” and “How do you evaluate what you have gained from volunteering? (Q5)”), and relatedness (e.g., “How do you evaluate the relationship with service recipients, teammates, and members of the voluntary service organization? (Q6)”). Regarding relatedness need satisfaction, for example, the above participants noted that some visitors who listened to their explanation would give them encouragement and support at the end of their presentation. At this point, the participants were asked to describe in detail the roles of visitors’ encouragement and support in the participants’ involvement in volunteering.

### 6.3. Data Analysis

All the interviews were audio-recorded and transcribed verbatim. Analysis of the qualitative data was guided by qualitative content analysis [48]. Similar to qualitative data analysis methods used by previous studies [4,44], two coders (i.e., the first author and a researcher unfamiliar with the study) individually coded and analyzed the interview data in Excel software (Microsoft, Redmond, WA, USA) through three steps (i.e., selecting the unit of analysis, creating categories, and establishing themes) based on the coding manual. The two coders had 98% agreement on selecting the unit of analysis. During selecting the unit of analysis, line-by-line coding was used to capture actions in each segment of the transcripts. And the transcripts were separated into condensed meaning units. For example, a volunteer stated, “In volunteer activities, we can plan the details of the teaching activities and communicate with local teachers and students independently (Participant 1′s answer to Q3)”. The answer was labeled as “feelings of high autonomy”. After the first step, creating categories helped us categorize and synthesize the condensed meaning units. For example, the “feelings of high autonomy” formed in the first step were classified as “autonomy need satisfaction” in this stage. Finally, organizing the categories into more systematic themes was the step of establishing themes. Establishing themes helped the researcher identify the relationships between the themes. For example, “autonomy need satisfaction” was summarized as “BPNS”.

## 7. Results of Qualitative Phase

Overall, the 33 interviews generated nearly 5.5 h of data and approximately 132 pages of transcripts. According to the results, factors that promoted sustained volunteering including volunteers’ three BPNS (frequency: 92.66%), honor-related and environmental factors (7.34%, such as material rewards, school requirements, obtaining a volunteer service certificate, obtaining extra points in the evaluation of scholarship). Therefore, volunteers’ three BPNS were the most important factors in promoting sustained volunteering. The proportion of each need satisfaction in general need satisfaction was shown in Table 3 and Supplementary Table S3. Specifically, the results of the qualitative phase found that competence and relatedness need satisfaction were most frequently mentioned by participants, which ultimately promoted their sustained volunteering. Besides, although autonomy need satisfaction was rarely mentioned, it was indispensable in promoting sustained volunteering.

### 7.1. Emphasizing the Satisfaction of Competence and Relatedness Needs

Among the factors that promoted sustained volunteering, the frequency of competence and relatedness needs satisfaction composed approximately 47.25% and 30.73% of general need satisfaction, respectively. As indicated by the results of the quantitative phase, competence need satisfaction was positively related to objective (including service length and service frequency) and subjective indicators of sustained volunteering. Throughout all the interviews, most of the volunteers described building a sense of competency when they gained knowledge, developed new skills (such as communication, teaching), or gained new experience from volunteering (17.89%). To be more specific, one of the participants, supporting this finding, stated, “volunteer teaching activities are helpful to my skills improvement, career knowledge, and experience (Participant 7)”. Volunteers also gained sense of value or meaning (15.14%), and a sense of accomplishment and competence (11.93%) from volunteering. Several participants made comments similar to the sentences such as “when I become a volunteer teacher in a rural area, I feel what I do is valuable (e.g., Participant 13, 24, 27, 30, and 33)”. Additionally, experiencing a challenge in difficult tasks (2.29%) in volunteering also prompted volunteers to continue.

Notably, except for competence need, volunteers experienced the satisfaction of the need for relatedness (30.73%) and associated it with sustained volunteering. The results of the quantitative phase found that relatedness need satisfaction could promote subjective sustained volunteering (e.g., increasing the intent to continue volunteering). In the interview, many participants mentioned that building relationships with others (such as service recipients, teammates, members of voluntary service organizations, 19.72%) could promote sustained volunteering. Positive peer relationships (5.50%) also made volunteers continue. Specifically speaking, some participants (e.g., Participant 6, 9, 10, 19, 25, and 32) made statements such as “I would like to continue volunteering if my friend could participate together with me.” (i.e., accompanied by friends, 1.38%). Teamwork (0.92%) and a sense of belonging (3.21%) to a team were also factors that promoted sustained volunteering.

Interestingly, the latent profile analyses identified one distinct group: low autonomy-high competence and relatedness group, which had higher sustained volunteering than other groups. The results of the qualitative phase also verified the unique role of competence and relatedness needs satisfaction in sustained volunteering. In short, for Chinese college student volunteers, the satisfaction of their competence and relatedness needs in volunteering was the key factor to promote sustained volunteering.

### 7.2. Low but Indispensable Autonomy Need Satisfaction

When volunteers were asked why they continued volunteering, a minority (14.68%) reported feeling a sense of autonomy. Factors that contributed to this were the voluntariness (not required) of participation in volunteer service (6.42%) and feelings of high autonomy in volunteering (8.26%). One of the volunteers noted, “The volunteering in the science and technology museum was quite autonomous. Generally, there was no one telling me what to do, so I would like to continue to be a volunteer in the museum (Participant 1)”. Among the three needs in promoting sustained volunteering, the proportion of autonomy need satisfaction was relatively low but indispensable. More precisely, in the quantitative phase, we found that the high group scored higher on sustained volunteering than other groups with low levels of BPNS. That is, it is optimal to simultaneously promote the satisfaction of volunteers’ three basic psychological needs (including autonomy need) to a higher degree. In the qualitative phase, volunteers also mentioned some factors that thwarted autonomy need, such as mandatory participation, lack of opportunities to express their opinions in volunteering and feelings of low autonomy. When the need for autonomy was frustrated, the volunteers’ intention to quit increased.

## 8. Discussion

In recent years, maintaining the stability and sustainability of volunteers has become one of the core issues in volunteer behavior, and it is directly related to the quality of voluntary service and the maintenance of the spirit of volunteerism [10,49]. Due to the importance of retaining volunteers, the present study investigated the role of the three BPNS in sustained volunteering via a mixed-methods design.

### 8.1. Three BPNS and Sustained Volunteering: From Variable- and Person-Centered Perspective

SDT stipulates that satisfaction of basic psychological need is essential for the generation of vitality, which can constructively regulate one’s behavior [50]. First, because earlier research has often examined the impact of three BPNS as a whole, without differentiating the individual role of the three needs [24], in the quantitative phase, we studied the three needs respectively and analyzed each need’s unique contributions to sustained volunteering using the variable-centered analyses. Consistent with previous studies based on SDT theory [22,28], the results revealed that satisfaction of competence and relatedness needs can significantly enhance sustained volunteering. The results of the qualitative also proved once again that volunteers who experienced high levels of satisfaction of the needs for competence (45.58%) and relatedness (27.43%) have a high level of sustained volunteering. In addition, contrary to the findings of Kim et al. [23] and Boezeman and Ellemers [12], the association between autonomy need satisfaction and sustained volunteering was not significant. Similarly, the frequency of autonomy need satisfaction (11.06%) was the lowest among the results of the quantitative phase. One of the reasons may be that it is more important for Chinese college students to learn skills and develop a sense of self-based accomplishment in volunteering to enhance their competence in the context of future employment pressure. Furthermore, in the Chinese context, especially Confucian culture, establishing positive relationships with others in volunteering is more in line with the characteristics of Chinese interpersonal communication. Ultimately, the variable-centered analyses demonstrated that competence and relatedness need satisfaction were stronger predictors than the satisfaction of the need for autonomy.

Second, in the quantitative phase, five BPNS profiles were identified in person-centered analyses: low (Profile 1), relatively low (Profile 2), moderate (Profile 3), low autonomy-high competence and relatedness (Profile 4), and high (Profile 5). These combinations indicated that the associations among the three BPNS were more complex. Among the five profiles, we found four common groups, that is, volunteers reported low, relatively low, moderate, and high levels of three BPNS. These profiles correspond to the results of previous studies [31,38] where similar morphology of profiles were identified. In addition to these common profiles, we also found a special profile (Profile 4) of volunteers, characterized by low levels of autonomy and high levels of competence and relatedness. Although Profile 4 accounted for only 7% of the total sample, the results showed no significant difference in the scores on sustained volunteering between profile 4 and profile 5. And the scores of these two profiles were higher than those of other profiles. The results indicated that satisfaction of high levels of competence and relatedness obtained through volunteering played critical roles in sustained volunteering.

### 8.2. An Integrated Perspective of Quantitative and Qualitative Stages

Based on SDT, we integrate the results of the quantitative and qualitative phase, which suggests that satisfaction of three basic psychological needs (especially competence and relatedness needs satisfaction) is critical in understanding volunteer behavior. Contexts supportive of three BPNS engender greater levels of intrinsic motivation [50,51]. According to the theory of organismic integration, sustained volunteering is a planned behavior that can be internally motivated [15]. Previous research has also found that volunteers have higher levels of satisfaction and likelihood of volunteering again when their key needs (e.g., competence and relatedness) are highly satisfied. This suggests the importance of volunteers’ sense of effectiveness, mastery, and social connection in volunteering. For competence need satisfaction, the findings of the current study converge with those of previous studies among undergraduate students, which showed that experiences of efficacy-building provided volunteers with confidence and motivation to continue their volunteer work [51]. As mentioned by Son and Wilson [52], volunteering enhances an individual’s feeling of personal control (mastery). That is, volunteering affords individuals the opportunity to gain a sense of accomplishment and competence, acquire new skills, and gain new experience or knowledge to satisfy the competence need, which eventually increases their future volunteer intentions.

For relatedness need satisfaction, the process of connecting with others is the process of reducing social isolation and strengthening social interaction. The findings support Hyde et al. [3], who found that a sense of social connection (e.g., spending time with friends) was essential for retaining novice volunteers, and volunteers in the sustained phases had a greater number of close connections (e.g., partners) than those in the novice and the transition phases. Especially for college students living in a collectivist culture that places more emphasis on social harmony, it is understandable that they emphasize the necessity of the relatedness dimension [53]. In addition, from the results of the qualitative phase, to some extent, freedom of choice and high autonomy also led volunteers to be more engaged in volunteering and less willing to quit volunteer organizations, which is consistent with prior research [13].

Overall, given volunteers’ high turnover rates, the results yield practical implications for the construction of a long-term mechanism of volunteering by expanding SDT. Recognizing that volunteers often engage in their work in nonprofit organizations with varying BPNS, volunteer organizations should pay attention to design activities that allow volunteers to fulfill their three BPNS, especially the competence and relatedness dimension. For instance, enabling volunteers to gain a sense of effectiveness and mastery (competence), build closeness and connection with significant others (relatedness), and experience choice and psychological freedom (autonomy).

## 9. Limitations and Future Research

Several limitations of the current study need to be considered. Firstly, the self-rating measures that we used could have been influenced by volunteers’ social desirability and self-report biases. Future research should use more objective data, such as supervisors’ reports. Secondly, because the current study was cross-sectional, we could not conclude the direction of effects between the three BPNS and sustained volunteering, and little was known about how these associations would unfold over time. Future research should also have a longitudinal design to prove associations between the three BPNS and sustained volunteering. Thirdly, the gender distribution of the sample was not balanced. Future studies should pay attention to gender sampling balance. Finally, recent evidence suggests that there are cross-cultural differences in the roles of three BPNS in promoting achievements [54], and the current study also finds the different roles of three BPNS in sustained volunteering among Chinese college student volunteers. This suggests that the three BPNS may play different roles in different activities and different cultural backgrounds. Therefore, future research should further explore the relationships between the three BPNS and the mechanism of the three BPNS in more diverse samples and cultural backgrounds.

## 10. Conclusions

By extending SDT, our proposed model provided a more comprehensive understanding of sustained volunteering. The current research explored the roles of the three BPNS in sustained volunteering through a mixed-methods design (i.e., quantitative and qualitative approaches). In the quantitative phase, a combined methodological approach was used. The results of variable-centered analyses revealed that only relatedness and competence need satisfaction were positively related to sustained volunteering. From the results of person-centered analyses, five different BPNS profiles among college volunteers were identified and linked in different ways to sustained volunteering. More importantly, volunteers in Profile 4 (low autonomy-high competence and relatedness group) and Profile 5 (high group) reported higher sustained volunteering than those in other profiles. The subsequent qualitative findings provided important insights, which could help illuminate the different roles of the three BPNS in sustained volunteering. The findings extend previous literature by offering a comprehensive perspective to understand the complex mechanism linking the three BPNS and long-term volunteering. Volunteer organizations and supervisors could use our findings to increase volunteers’ three BPNS and facilitate volunteer retention in future activities.

## Figures and Tables

**Table 1:** Summary of the model estimates for the regression analysis of sustained volunteering.

Variables	Service Length		Service Frequency		Objective ^a^		Subjective ^b^	
	*β*	*t*	*β*	*t*	*β*	*t*	*β*	*t*
Gender	0.08	1.01	−0.06	−0.75	0.01	0.14	0.13	1.85
Age	−0.11	−5.00 ***	−0.10	−4.43 ***	−0.10	−5.43 ***	0.01	0.49
Autonomy	−0.01	−0.33	−0.01	−0.19	−0.01	−0.30	−0.02	−0.74
Relatedness	0.08	0.90	−0.03	−0.33	0.02	0.32	0.41	5.39 ***
Competence	0.21	2.73 **	0.23	2.99 **	0.22	3.29 **	0.34	4.93 ***
R^2^	0.07 ***		0.05 ***		0.07 ***		0.25 ***	
Adjusted R^2^	0.06 ***		0.04 ***		0.07 ***		0.25 ***	
F	11.45 ***		7.60 ***		12.16 ***		53.01 ***	

*Note.* ^a b^ Objective and subjective indicators of sustained volunteering. ** *p* < 0.01; *** *p* < 0.001.

**Table 2:** Differences in mean scores for the three basic psychological need satisfaction (BPNS) and sustained volunteering.

Variables	Profile 1 (a)		Profile 2 (b)		Profile 3 (c)		Profile 4 (d)		Profile 5 (e)		*F*	*η_p_* ^2^
	*M*	*SD*	*M*	*SD*	*M*	*SD*	*M*	*SD*	*M*	*SD*		
Profile variables												
Autonomy	−0.68 ^bcde^	0.78	−0.26 ^ade^	0.82	−0.07 ^ade^	0.90	−1.29 ^abce^	0.62	0.88 ^abcd^	0.67	106.79 ***	0.35
Relatedness	−2.29 ^bcde^	0.46	−1.13 ^acde^	0.37	0.02 ^abde^	0.36	1.08 ^abc^	0.29	1.07 ^abc^	0.32	1423.16 ***	0.71
Competence	−1.54 ^bcde^	0.66	−1.09 ^acde^	0.55	−0.06 ^abde^	0.56	0.90 ^abc^	0.54	1.08 ^abc^	0.49	482.82 ***	0.88
Outcome variables												
Service length	62.16 ^d^	66.67	59.94 ^de^	57.36	72.74 ^d^	62.85	99.00 ^abc^	70.37	86.24 ^b^	67.37	6.54 ***	0.03
Service frequency	2.70	1.33	2.71 ^e^	1.11	2.88	1.21	3.17	1.29	3.07 ^b^	1.37	2.89 *	0.01
Objective	−0.18	0.93	−0.19 ^de^	0.76	−0.02	0.85	0.29 ^b^	0.94	0.16 ^b^	0.94	5.92 ***	0.03
Subjective	4.65 ^bcde^	1.12	5.35 ^acde^	1.00	5.90 ^abde^	0.83	6.42 ^abc^	0.75	6.33 ^abc^	0.89	50.37 ***	0.20

*Note.* Scores for three BPNS were standardized. For the three BPNS and sustained volunteering, superscripts (e.g., −0.68 ^bcde^) indicated that, based on Tukey post hoc tests, there was a significant difference between the autonomy scores of profile 1 and profiles 2, 3, 4 and 5 (*p* < 0.05). *η_p_*^2^ = partial eta squared. * *p* < 0.05; *** *p* < 0.001.

**Table 3:** Codes and frequencies of volunteers’ BPNS.

Categories and Subcategories	Frequencies of Categories	Frequencies of Subcategories
Category 1: Autonomy need satisfaction	14.68%	
voluntary (not required) participation in volunteer service		6.42%
feelings of high autonomy		8.26%
Category 2: Competence need satisfaction	47.25%	
acquire new knowledge and experience/develop skills		17.89%
a sense of value/meaning		15.14%
a sense of accomplishment and competence		11.93%
experience a challenge in difficult tasks		2.29%
Category 3: Relatedness need satisfaction	30.73%	
build relationships with others (such as recipients, teammates, members of voluntary service organizations)		19.72%
positive volunteer peer relationships		5.50%
a sense of belonging		3.21%
accompanied by friends		1.38%
teamwork		0.92%

## Data Availability

The data are not publicly available to protect the confidentiality of the participants.

## Supplementary Materials

The followings are available online at https://www.mdpi.com/article/10.3390/ijerph182413229/s1. Table S1: Descriptive statistics and correlation matrix of BPNS and sustained volunteering; Table S2: Fit indices for latent profile analysis of volunteers’ BPNS; Table S3: Codes and frequencies of volunteers’ BPNS.
