# Supplementary material for: Does Basic Psychological Need Satisfaction Matter to College Students’ Sustained Volunteering? A Mixed-Methods Study
Source: Int J Environ Res Public Health. 2021 Dec 15;18(24):13229. doi: 10.3390/ijerph182413229 (PMC8701654; doi:10.3390/ijerph182413229)
Supplement: Supplementary file 1 [file ijerph-18-13229-s001.zip › ijerph-1480334-supplementary.pdf]

Supplementary tables

Table S1. Means (M), standard deviations (SD), and correlation matrix of BPNS and sustained volunteering.

Variables	1	2	3	4	5	6	7	8	9	10
1 Gender	–									
2 Age	−0.08*	–								
3 Autonomy	−0.15**	0.02	–							
4 Relatedness	0.02	0.03	0.27**	–						
5 Competence	−0.01	0.05	0.24**	0.80**	–					
6 General need satisfaction [a]	−0.08*	0.04	0.76**	0.79**	0.78**	–				
7 Service length	0.05	−0.17**	0.03	0.17**	0.19**	0.15**	–			
8 Service frequency	−0.01	−0.14**	0.03	0.11**	0.15**	0.11**	0.53**	–		
9 Objective [b]	0.02	−0.18**	0.03	0.16**	0.19**	0.14**	0.88**	0.88**	–	
10 Subjective [c]	0.06	0.03	0.09**	0.47**	0.46**	0.39**	0.18**	0.23**	0.23	–
M	1.73	21.61	4.94	6.05	5.72	5.57	74.73	2.90	0.00	5.86
SD	0.44	1.63	1.14	0.67	0.74	0.66	64.49	1.25	0.88	1.00

Note. [a] General need satisfaction was calculated as the average of the three need satisfactions. [b] [c] Objective and subjective indicators of sustained volunteering. *$p < 0.05$; **$p < 0.01$. The same applies below.

Table S2. Fit indices for latent profile analysis of volunteers' BPNS.

Number of profiles	AIC	BIC	aBIC	Entropy	AlMR LRT	BLRT
1	6845.45	6873.58	6854.52	–	–	–
2	6260.90	6307.78	6276.03	0.77	-3416.72***	-3416.72***
3	5970.31	6035.94	5991.48	0.82	-3120.45***	-3120.45***
4	5896.77	5981.16	5924.00	0.85	-2971.15	-2971.15***
5	5861.93	5965.07	5895.21	0.82	-2930.38*	-2930.38***

Table S3. Codes and frequencies of volunteers' BPNS.

Categories and subcategories	Exemplar quotes	Frequencies of categories	Frequencies of subcategories
**Category 1: Autonomy need satisfaction**			
voluntary (not required) participation in volunteer service	"When I learned the information about helping the elderly in nursing homes, I decided to participate in volunteer activities. I'll continue to engage in such activities, which bring me great enjoyment." (P30)	14.68%	6.42%
feelings of high autonomy	"I like to volunteer at the Science and Technology Museum because I have more autonomy to do what I want to do."(P1)		8.26%
**Category 2: Competence need satisfaction**			
acquire new knowledge and experience/develop skills	"I continue to volunteer because it allows me to learn new knowledge."(P4, 24 and 29)	47.25%	17.89%
a sense of value/meaning	"When I became a rural volunteer teacher, I felt valuable." (P13, 24, 27 30 and 33)		15.14%
a sense of accomplishment and competence	"I did some substantial work such as serving athletes and editing, which gave me a sense of accomplishment."(P6)		11.93%

experience challenge in difficult tasks	"There will be some differences in serving these two different age groups, and I want to experience the challenge."(P33)		2.29%
**Category 3: Relatedness need satisfaction**			
building relationships with others (such as recipients, teammates, members of voluntary service organizations)	"When I was received by the recipient (e.g., a child) with friendliness, I felt happy and less tired. This time, I really want to continue to communicate with her and to serve others."(P1)		19.72%
positive volunteer peer relationships	"The positive interaction between volunteer peers could encourage us to continue volunteering. And by communicating with each other, we could expand our thinking."(P9)	30.73%	5.50%
a sense of belonging	"Our team leader did a good job. He gave us regular trainings, organized meetings and team building activities, etc. Therefore, the cohesion of our team is quite strong."(P25)		3.21%
accompanied by friends	"I would like to continue volunteering if my friend could participate together with me."(P6, 9, 10, 19, 25 and 32)		1.38%
teamwork	"In fact, it's more efficient to work with peers."(P6 and 27)		0.92%

Note. P = Participant, P1 represents the participant coded as 1.
